# Supplementary material for: Tuning ferroelectric phase transition temperature by enantiomer fraction
Source: Nat Commun. 2024 Feb 17;15:1464. doi: 10.1038/s41467-024-45986-6 (PMC10874439; doi:10.1038/s41467-024-45986-6)
Supplement: Supplementary file 1 — Supplementary Information [file 41467_2024_45986_MOESM1_ESM.pdf]

Supporting Information

Tuning Ferroelectric Phase Transition Temperature by Enantiomer Fraction

Fan et al.

Contents

Supplementary Fig. 1 | PXRD patterns of **1*Rac*** and **1*S*** at 293 K.

Supplementary Fig. 2 | TGA curves of **1*Rac*** and **1*S***.

Supplementary Fig. 3 | SHG strengths of **1*Rac*** and KDP at 293 K.

Supplementary Fig. 4 | Structure of **1*Rac***.

Supplementary Fig. 5 | (a) 3D d_{norm} surface and (b-d) 2D fingerprint plots of **1*Rac*** at 293 K.

Supplementary Fig. 6 | N–H···Br interactions between the organic cations and inorganic layers of ***1Rac*** at 293 K.

Supplementary Fig. 7 | (a) 3D d_{norm} surface and (b-d) 2D fingerprint plots of **1*Rac*** at 370 K.

Supplementary Fig. 8 | Structure and phase transition properties.

Supplementary Fig. 9 | Properties of **1*Rac*** synthesized by solution and solid-phase synthesis (SPS), respectively.

Supplementary Fig. 10 | PXRD study of (S-3AMP)$_x$(R-3AMP)$_{1-x}$PbBr$_4$ (x = 0.5 – 1) at 293 K.

Supplementary Fig. 11 | Properties of physical mixture of **1*Rac*** and **1*S*** at 293 K.

Supplementary Fig. 12 | Real part of the dielectric constant of (S-3AMP)$_x$(R-3AMP)$_{1-x}$PbBr$_4$ (x = 0.5 – 1).

Supplementary Fig. 13 | Structure and phase transition properties of (S-3AMP)$_x$(R-3AMP)$_{1-x}$PbBr$_4$.

Supplementary Fig. 14 | SHG studies of (S-3AMP)$_x$(R-3AMP)$_{1-x}$PbBr$_4$ (x = 0.5 – 1) at 293 K.

Supplementary Fig. 15 | PXRD study of (S-3AMP)$_x$(R-3AMP)$_{1-x}$PbBr$_4$ (x = 0.5 – 1) at (a) 293 K and (b) 440 K.

Supplementary Fig. 16 | Semiconducting properties of **1*Rac***.

Supplementary Fig. 17 | Circularly polarization light excited PL spectroscopy at 298 K.

Supplementary Fig. 18 | PL spectral of (S-3AMP)$_x$(R-3AMP)$_{1-x}$PbBr$_4$ (x = 0.5 – 1) at 298 K.

Supplementary Fig. 19 | PL decay curves of the synthesized materials at 298 K.

Supplementary Table 1 | Crystallographic data and structural refinement details of **1*Rac***.

Supplementary Table 2 | Selected hydrogen bonds of **1*Rac*** (293 K).

Supplementary Table 3 | Bond Angles and Bond Length of **1*Rac***.

Supplementary Table 4. Polynomial fitting equations and fitting parameters.

Supplementary Table 5. Summary of phase transition temperature (T_C), enthalpy change (ΔH), entropy change (ΔS), and orientation number (N) of (S-3AMP)$_x$(R-3AMP)$_{1-x}$PbBr$_4$ (x = 0.5 – 1).

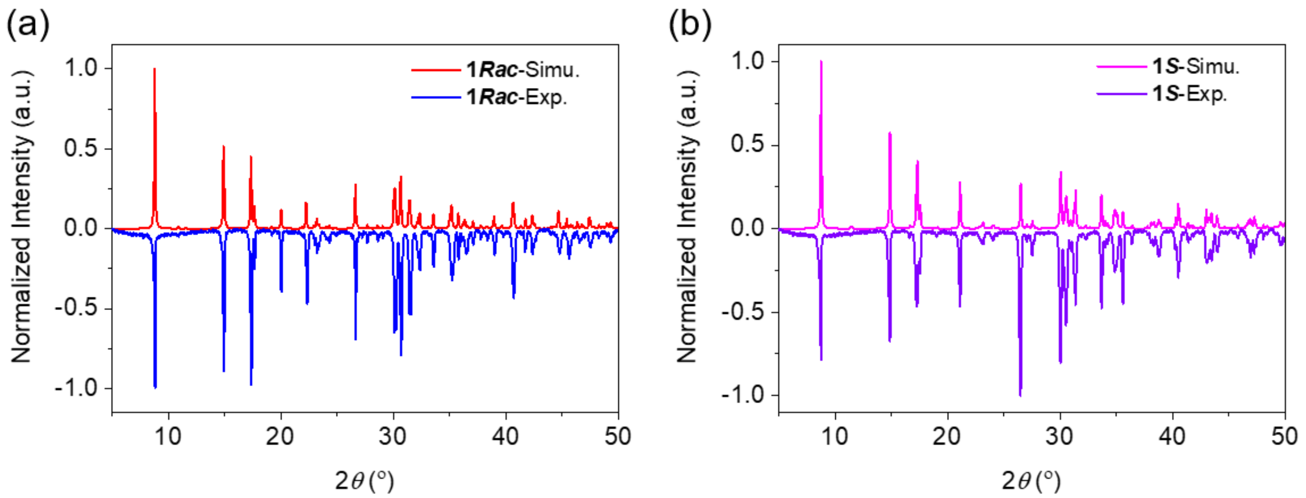

Supplementary Fig. 1 | PXRD patterns of **1*Rac*** and **1*S*** at 293 K.

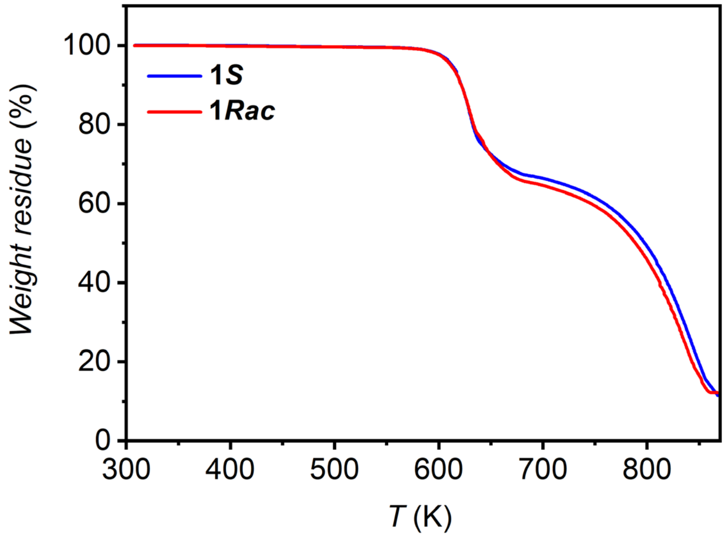

Supplementary Fig. 2 | TGA curves of **1*Rac*** and **1*S***.

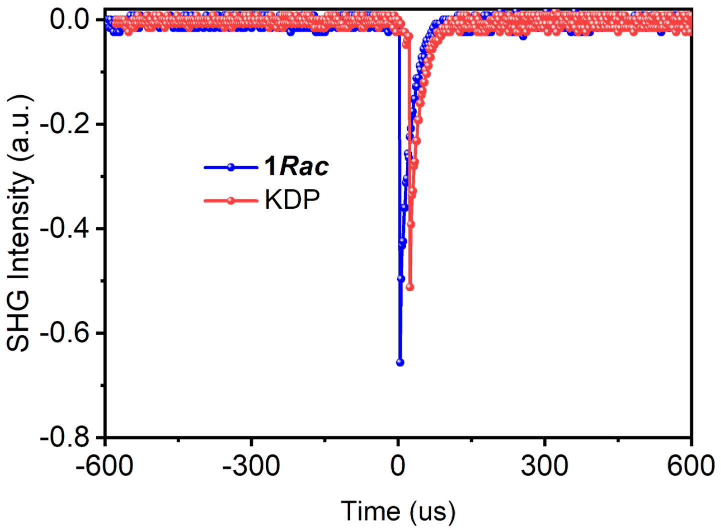

Supplementary Fig. 3 | SHG strengths of **1*Rac*** and KDP at 293 K.

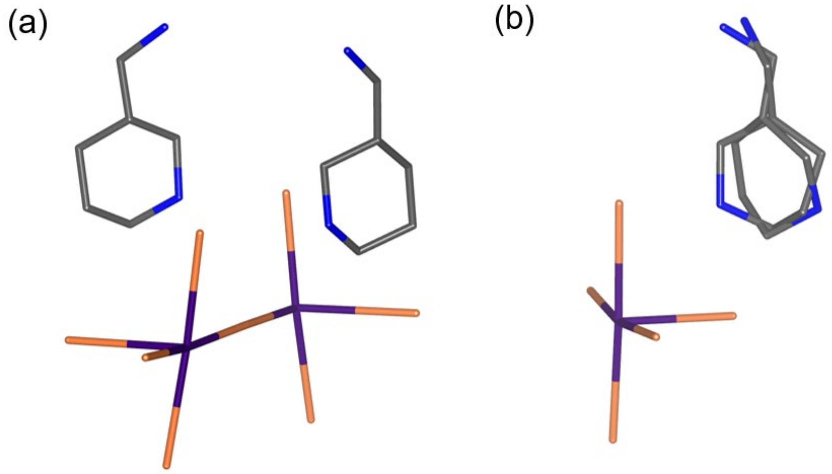

Supplementary Fig. 4 | Structure of 1*Rac*. Asymmetric unit of **1*Rac*** at (a) 293 K and (b) 370 K, showing Pb(II) coordination environment. The 3AMP cation in (b) is disordered. H atoms are omitted for clarity.

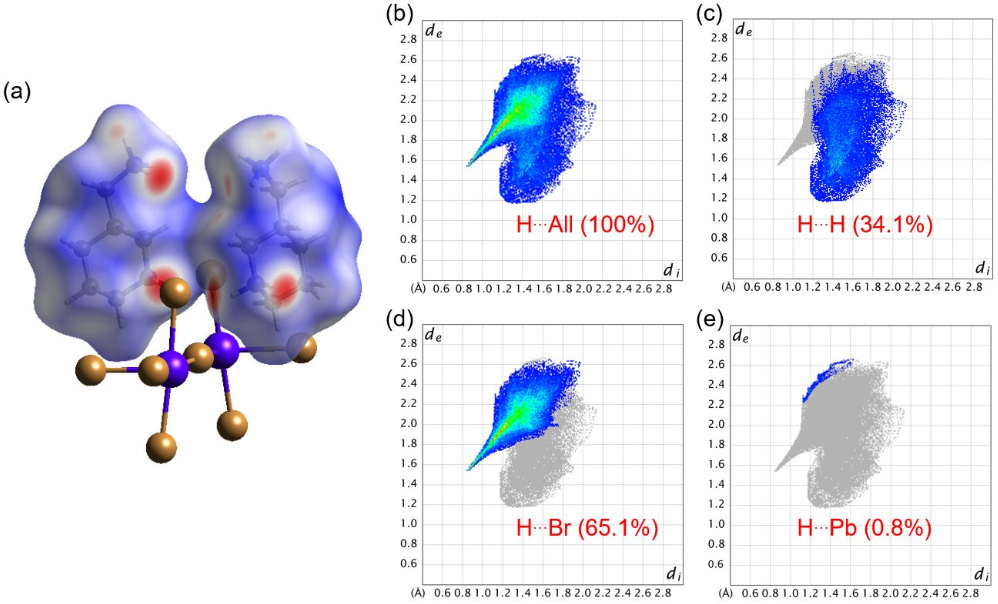

Supplementary Fig. 5 | (a) 3D d_{norm} surface and (b-d) 2D fingerprint plots of **1*Rac*** at 293 K. Red, white, and blue regions of the Hirshfeld surfaces indicate positive (close contact), neutral, and negative isoenergies, respectively.

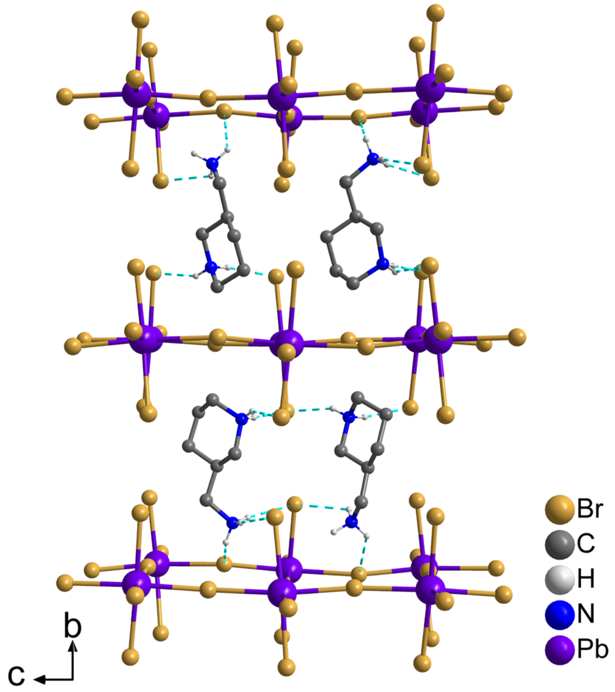

Supplementary Fig. 6 | N–H···Br interactions between the organic cation and inorganic layer of ***1Rac*** at 293 K.

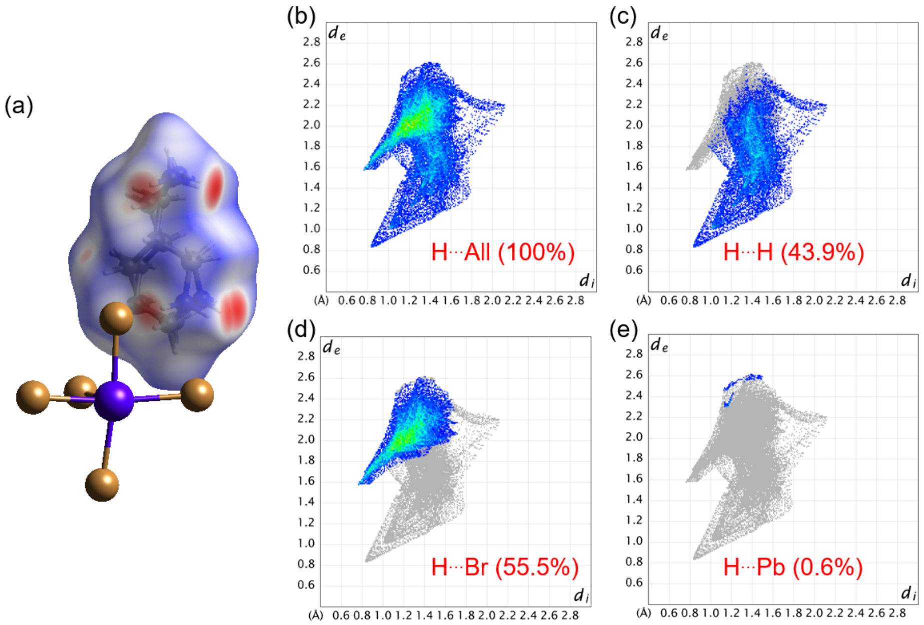

Supplementary Fig. 7 | (a) 3D d_{norm} surface and (b-d) 2D fingerprint plots of **1*Rac*** at 370 K. Red, white, and blue regions of the Hirshfeld surfaces indicate positive (close contact), neutral, and negative isoenergies, respectively.

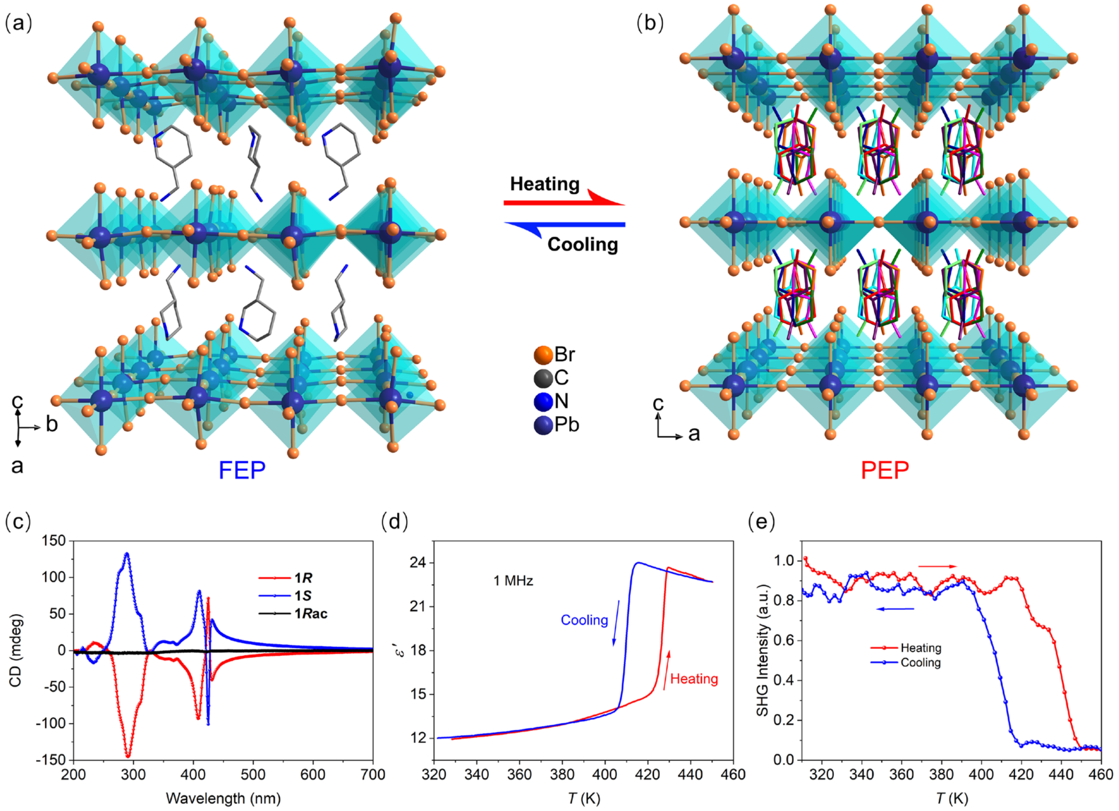

Supplementary Fig. 8 | Structure and phase transition properties. Crystal structures of **1*S*** (a) in the ordered FEP and (b) in the disordered PEP. Hydrogen atoms are omitted for clarity. (c) Chiroptical properties of **1*R/S*** and **1*Rac*** films. (d) Dielectric transition of **1*S***. (e) SHG signal of **1*S***.

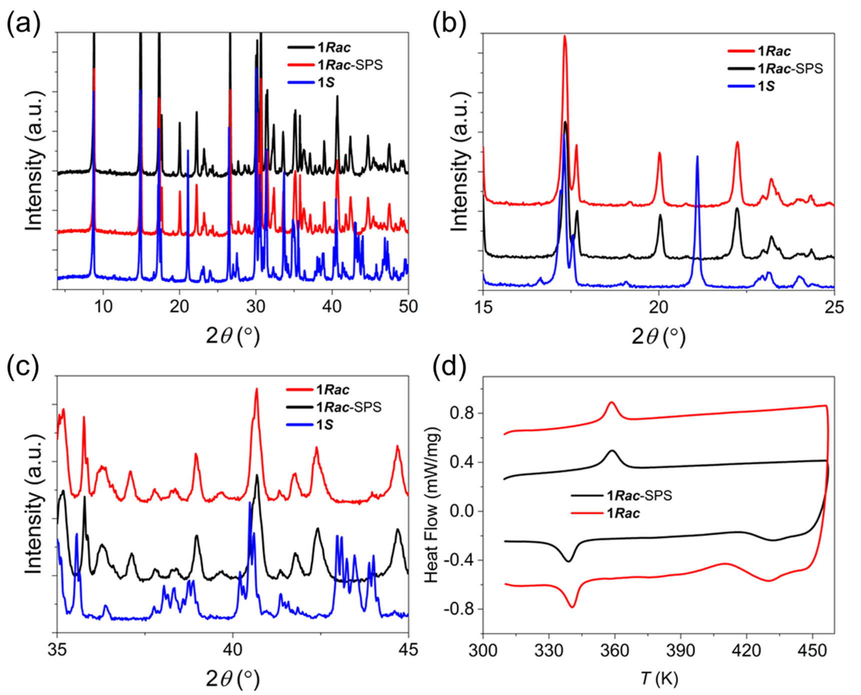

Supplementary Fig. 9 | Properties of 1*Rac* synthesized by solution and solid-phase synthesis (SPS), respectively. a-c PXRD patterns of **1*Rac*** synthesized by solution method and solid-phase synthesis method. **d** DSC comparison of **1*Rac*** synthesized by solution method and solid-phase synthesis method.

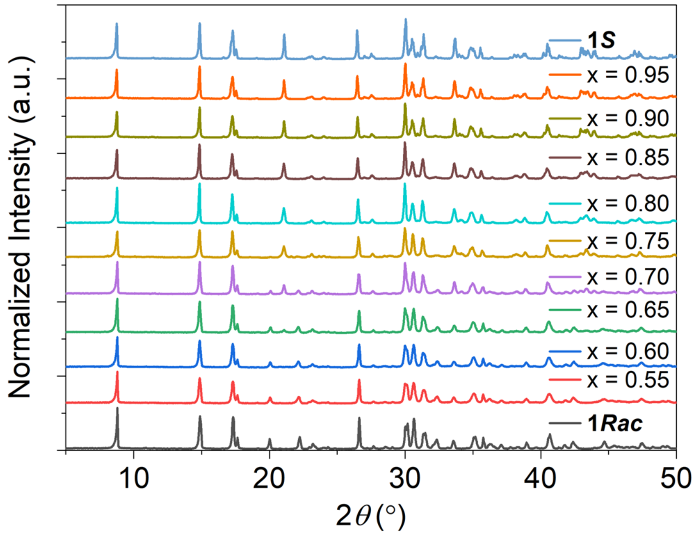

Supplementary Fig. 10 | PXRD study of (S-3AMP)$_x$(R-3AMP)$_{1-x}$PbBr$_4$ (x = 0.5 – 1) at 293 K.

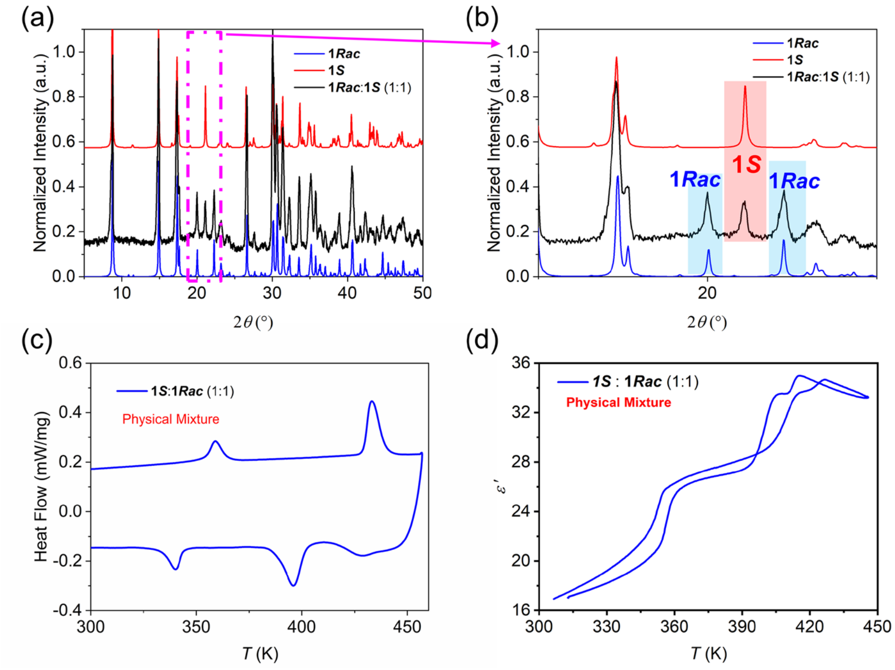

Supplementary Fig. 11 | Properties of physical mixture of 1*Rac* and 1*S* at 293 K. a PXRD patterns. **b** Enlarged PXRD patterns. **c** DSC curves. **d**. Real part of the dielectric constant.

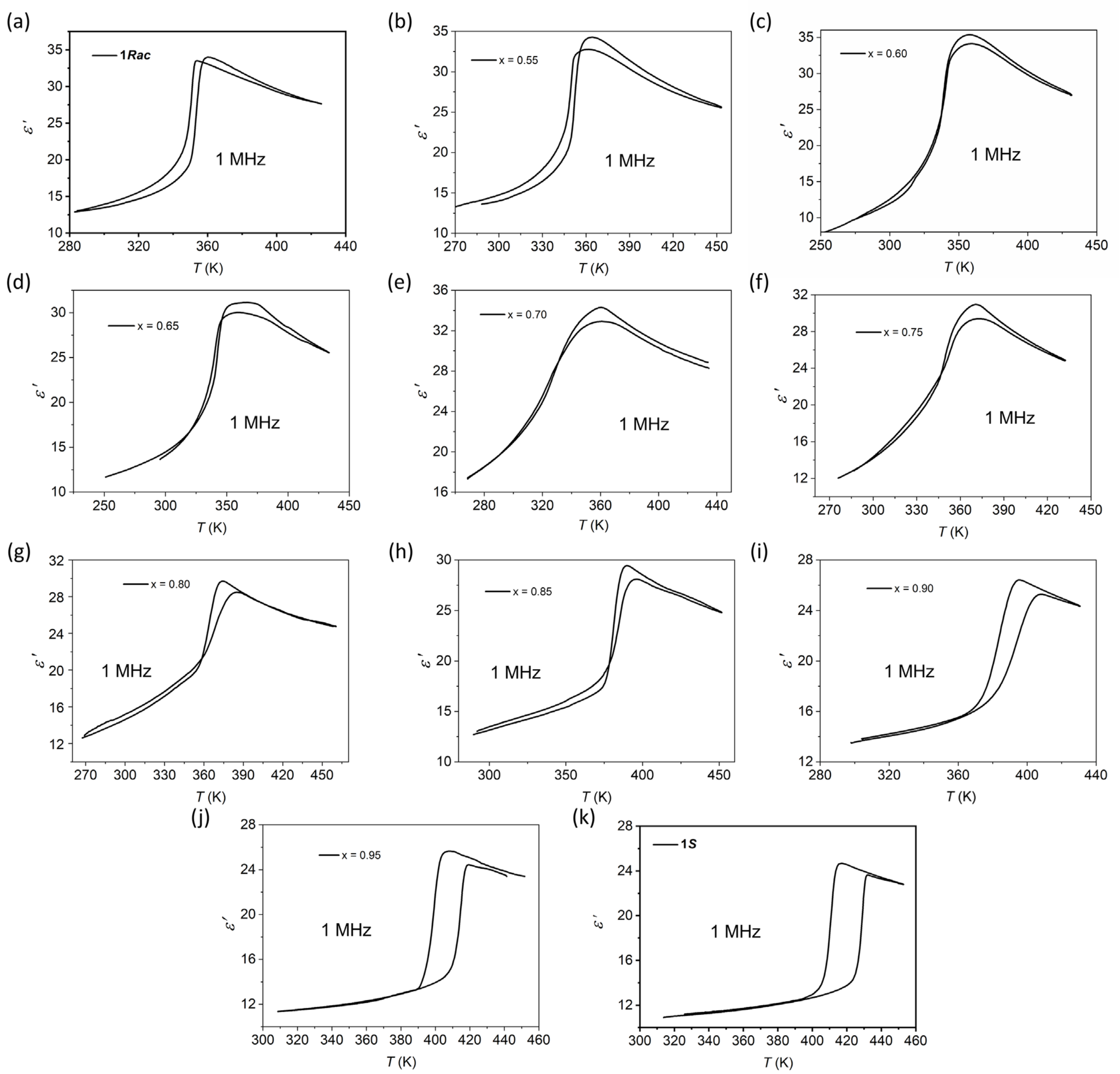

Supplementary Fig. 12 | Real part of dielectric constant of (S-3AMP)$_x$(R-3AMP)$_{1-x}$$PbBr_4$ (x = 0.5 – 1).

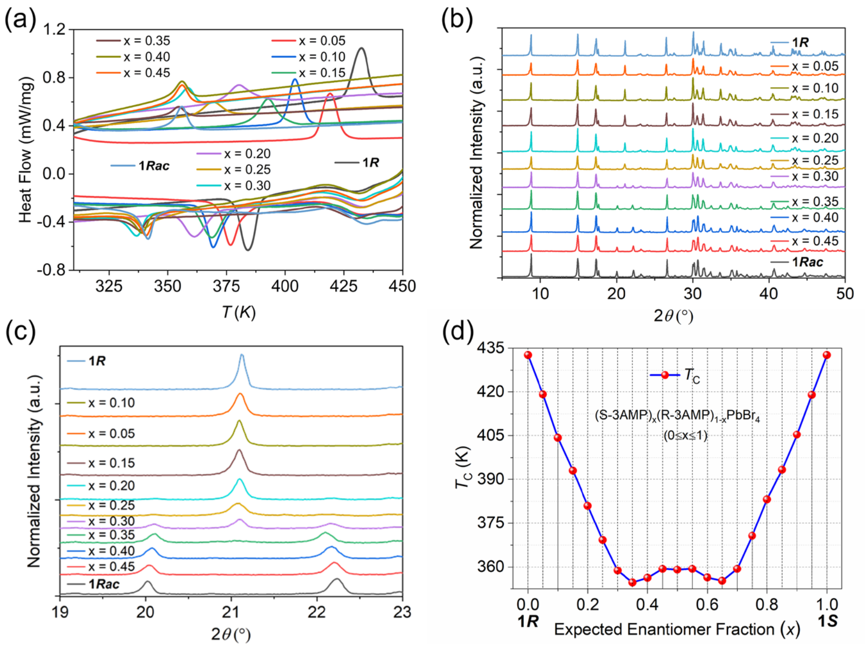

Supplementary Fig. 13 | Structure and phase transition properties of (*S*-3AMP)$_x$(*R*-3AMP)$_{1-x}$PbBr$_4$. a PXRD patterns of x = 0 – 0.5. **b** DSC curves of x = 0 – 0.5. **c** Nonlinear correlation between the phase transition temperature and x (0 – 0.5) **d**. Relationship between phase transition temperature and x during DSC heating process of (x = 0 – 1).

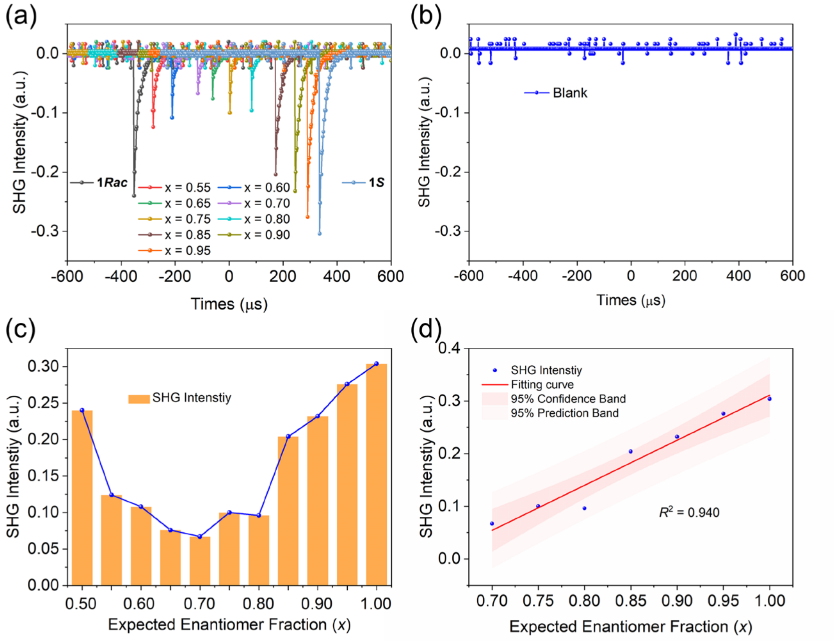

Supplementary Fig. 14 | SHG studies of (*S*-3AMP)$_x$(*R*-3AMP)$_{1-x}$PbBr$_4$ (x = 0.5 – 1) at 293 K. a SHG of the samples. **b** SHG of blank. **c** Comparison of the *P* values of different enantiomer fractions. **d**

Linear correlation between the SHG intensity and x. The solid lines fit a linear regression model with the corresponding R^2 value, 95% confidence band, and 95% prediction band (shaded region).

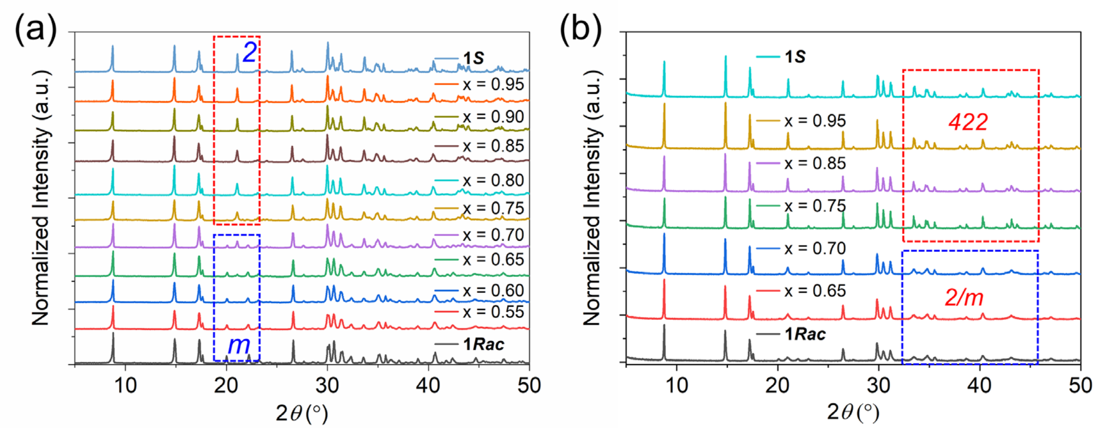

Supplementary Fig. 15 | PXRD patterns of (S-3AMP)$_x$(R-3AMP)$_{1-x}$$PbBr_4$ (x = 0.5 – 1) at (a) 293 K and (b) 440 K.

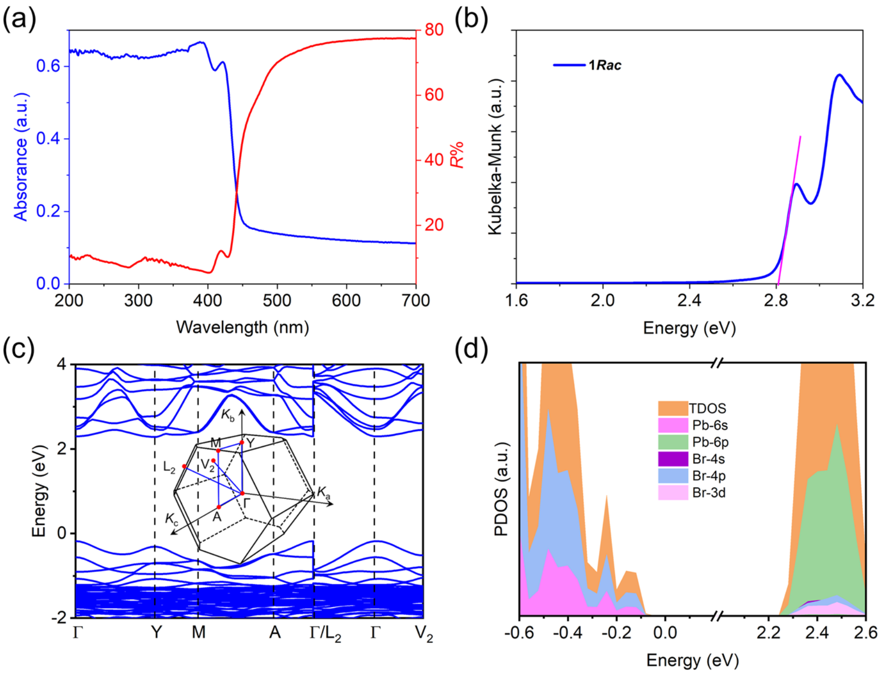

Supplementary Fig. 16 | Semiconducting properties of 1*Rac*. (a) UV−vis diffuse reflectance and absorption spectra of **1*Rac***. (b) Optical bandgap derived from Kubelka-Munk function based on diffuse reflection data. (c, d) DFT-PBE band structures and projected density of states (PDOS). Inset: Brillouin zone showing the k-path.

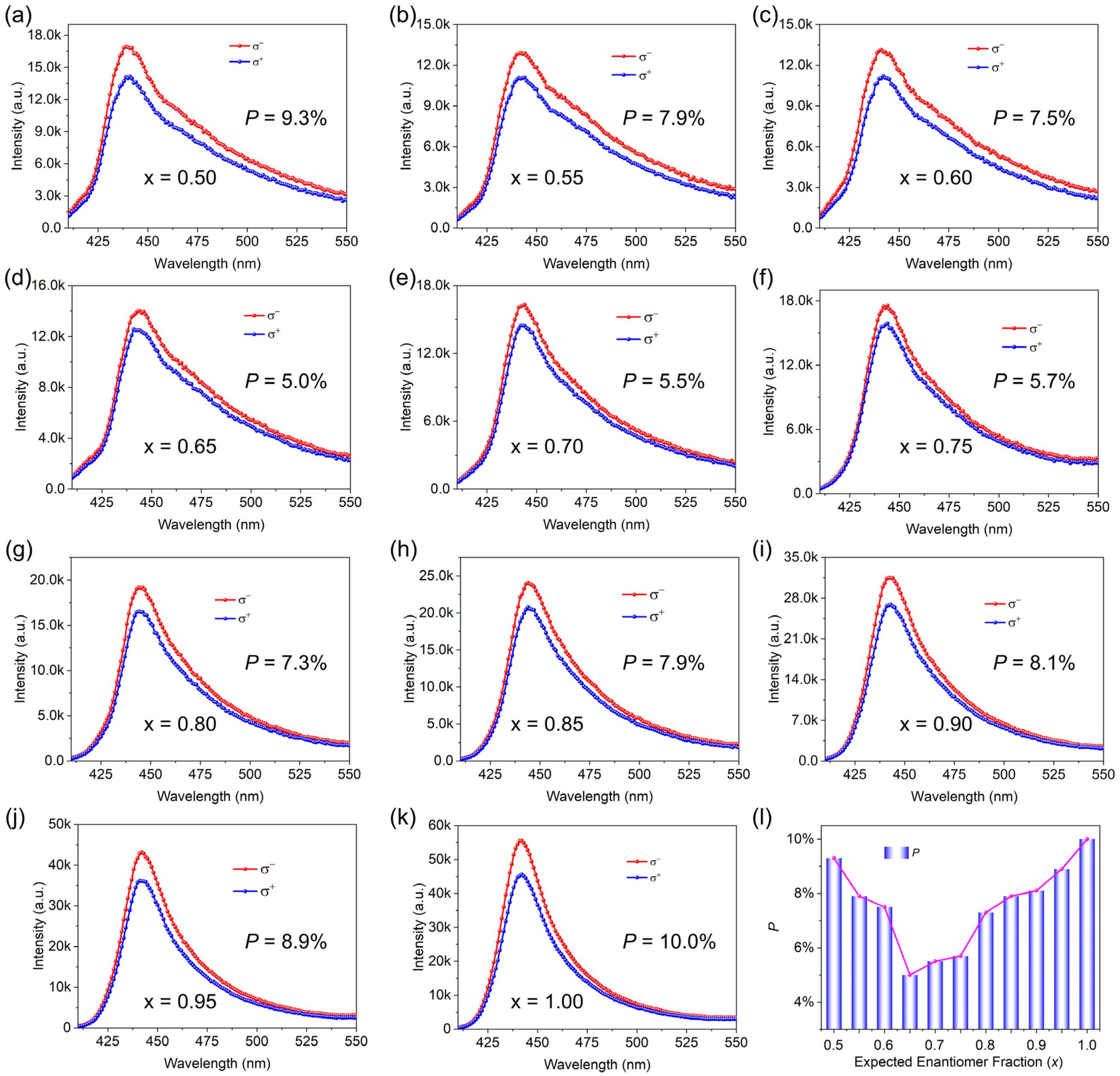

Supplementary Fig. 17 | Circularly polarization light excited spectroscopy at 298 K. a−k CPLEPL spectra of (*S*-3AMP)$_x$(*R*-3AMP)$_{1-x}$ (x = 0.5 − 1) upon L-CPL (σ^+) and R-CPL (σ^-) excitation at 395 nm and 298 K. **l** Comparison of the *P* values with different enantiomer fractions.

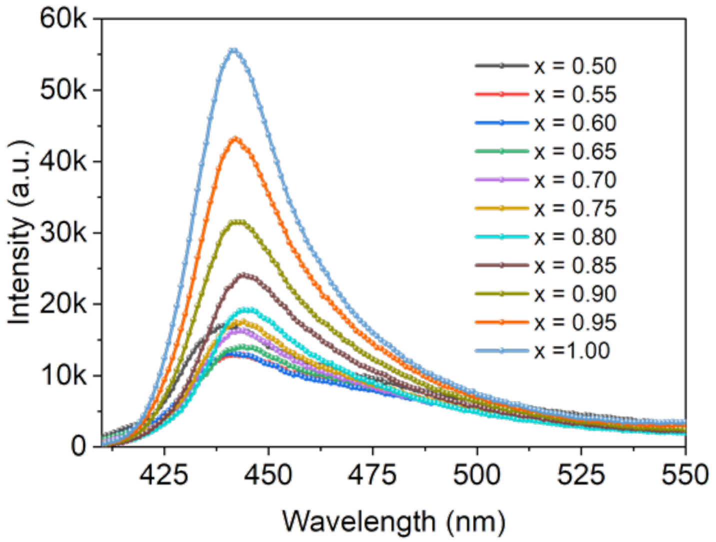

Supplementary Fig. 18 | PL spectra of $(S\text{-3AMP})_x(R\text{-3AMP})_{1-x}PbBr_4$ (x = 0.5 – 1) at 298 K.

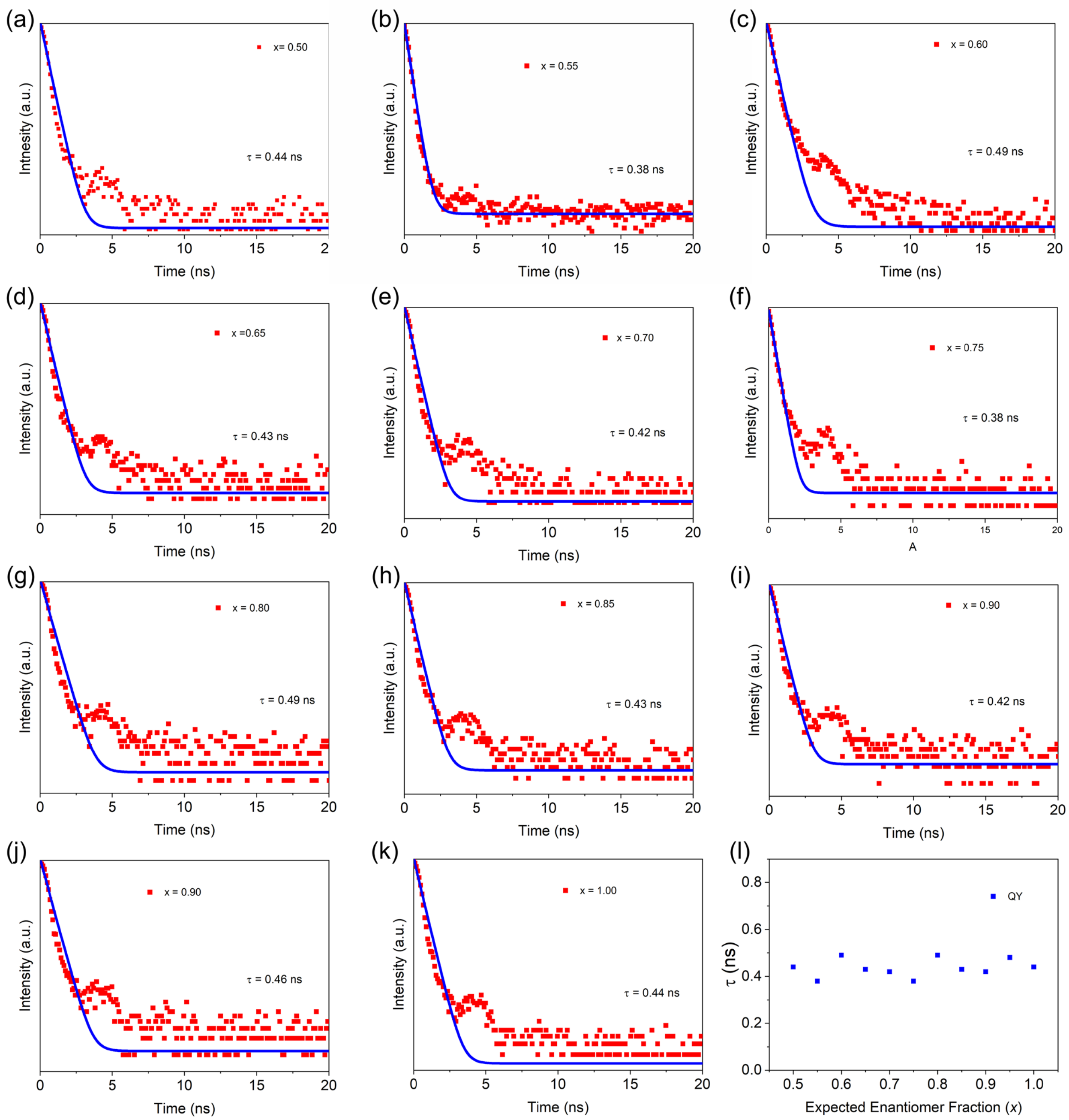

Supplementary Fig. 19 PL decay curves of the synthesized materials at 298 K. a−k PL decay curves of x = 0.5 − 1 at 298 K. **l** Comparison of the PL lifetime τ values with different enantiomer fractions.

Supplementary Table 1. Crystallographic data and refinement parameters of **1*Rac***.

	**1*Rac***	
	293 K	370 K
Formula	$C_{12}H_{32}N_4Pb_2Br_8$	$C_6H_{16}N_2Pb_1Br_4$
Formula weight	1286.02	643.04
Crystal system	Monoclinic	Monoclinic
space group	*Cc*	*C2/c*
a / Å	11.9049(5)	11.8979(16)
b / Å	20.0753(8)	20.148(2)
c / Å	11.9470(5)	11.9864(13)
α / º	90	90
β / º	95.940(4)	95.346(11)
γ / º	90	90
V / Å^3	2839.9(2)	2860.9(6)
Z	4	8
Flack parameter	0.276(16)	/
D_{calc} / g·cm^{-3}	3.008	2.986
μ / mm^{-1}	23.104	22.935
total reflns	4131	3137
obsd reflns (I > 2σ(I))	3700	2489
R_{int}	0.0264	0.0476
$R_1{}^a$ / $wR_2{}^b$ (I > 2σ(I))	0.0372, 0.0973	0.0568, 0.1262
R^1/wR^2 (all data)	0.0427, 0.0998	0.0764, 0.1416
GOF	1.07	1.08
$\Delta\rho^c$ / e·Å^{-3}	1.83/−2.41	2.35/−1.83

^{a} $R_1 = \Sigma| |F_o| - |F_c| | / |F_o|$. ^{b} $wR_2 = [\Sigma w(F_o^2 - F_c^2)^2] / \Sigma w(F_o^2)^2]^{1/2}$. ^{c} Maximum and minimum residual electron density.

Supplementary Table 2. Selected hydrogen bonds of **1*Rac*** (293 K).

D−H···A	D−H / Å	H···A / Å	D···A / Å	∠DHA / °
**1*R***				
N(4)−H(4D)···Br(5)	0.89	2.53	3.42(2)	178.3
N(4)−H(4C)···Br(1)	0.89	2.52	3.329(16)	151.8
N(3)−H(3E)···Br(8)^{i}	0.89	2.54	3.41(2)	167.9
N(3)−H(3D)···Br(2)^{ii}	0.89	2.88	3.43(3)	121.6
N(3)−H(3C)···Br(3)^{iii}	0.89	2.54	3.42(14)	170.8
N(2)−H(2B)···Br(5)	0.89	2.55	3.358(19)	151.2
N(2)−H(2A)···Br(8)^{iv}	0.89	2.58	3.46(15)	170.7
N(1)−H(1E)···Br(7)^{v}	0.89	3.00	3.82 (3)	154.6
N(1)−H(1E)···Br(2)^{iii}	0.89	2.91	3.42(2)	117.9
N(1)−H(1D)···Br(7)^{i}	0.89	3.12	3.94(3)	154.0

Symmetry codes: (i) x+1/2, y−1/2, z; (ii) x−1/2, −y+3/2, z+1/2; (iii) x−1/2, y−1/2, z; (iv) x, −y+2, z−1/2; (v) x+1/2, −y+3/2, z−1/2;

Supplementary Table 3. Bond angles and bond lengths of **1*R*ac**.

Bond Angles	Angle / °	Bond Lengths	Length / Å
**1*Rac***-293 K			
Pb(1)^{i} –Br(2)–Pb(1)	165.01(16)	Br(1)–Pb(1)	2.972(3)
Pb(1)–Br(4)–Pb(2)	175.30(18)	Br(2)–Pb(1)^{i}	2.894(4)
Pb(2)^{ii}–Br(6)–Pb(2)	167.27(16)	Br(2)–Pb(1)	3.134(4)
Pb(2)–Br(7)–Pb(1)^{iii}	167.92(8)	Br(3)–Pb(1)	3.057(3)
Br(2)^{ii}–Pb(1)–Br(1)	89.35(15)	Br(4)–Pb(1)	2.977(5)
Br(2)^{ii}–Pb(1)–Br(4)	90.20(15)	Br(4)–Pb(2)	3.044(5)
Br(1)–Pb(1)–Br(4)	86.53(10)	Br(5)–Pb(2)	2.971(3)
Br(2)^{ii}–Pb(1)–Br(7)^{iiii}	87.58(14)	Br(6)–Pb(2)^{ii}	2.931(3)
Br(1) –Pb(1)–Br(7)^{iiii}	85.39(10)	Br(7)–Pb(1)^{iii}	2.995(4)
Br(4)–Pb(1)–Br(7)^{iiii}	171.65(10)	Br(7)–Pb(2)	2.942(4)
Br(2)^{ii}–Pb(1)–Br(3)	88.99(12)	Br(8)–Pb(2)	3.064(3)
Br(1)–Pb(1)–Br(3)	170.53(13)		
Br(4)–Pb(1)–Br(3)	102.80(10)		
Br(7)^{iiii}–Pb(1)–Br(3)	85.22(10)		
Br(2)^{ii}–Pb(1)–Br(2)	166.28(16)		
Br(1)–Pb(1)–Br(2)	94.79(15)		
Br(4)–Pb(1)–Br(2)	103.08(13)		
Br(7)^{iiii}–Pb(1)–Br(2)	79.74(15)		
Br(3)–Pb(1)–Br(2)	84.77(12)		
Br(6)^{i}–Pb(2)–Br(7)	86.48(14)		
Br(6)^{i}–Pb(2)–Br(5)	90.55(13)		
Br(7)–Pb(2)–Br(5)	88.35(10)		
Br(6)^{i}–Pb(2)–Br(4)	85.80(15)		
Br(7)–Pb(2)–Br(4)	169.19(14)		
Br(5)–Pb(2)–Br(4)	84.16(11)		
Br(6)^{i}–Pb(2)–Br(8)	91.54(12)		
Br(7)–Pb(2)–Br(8)	88.96(9)		
Br(5)–Pb(2)–Br(8)	176.49(5)		
Br(4)–Pb(2)–Br(8)	98.80(8)		
Br(6)^{i}–Pb(2)–Br(6)	166.91(16)		
Br(7)–Pb(2)–Br(6)	81.77(15)		
Br(5)–Pb(2)–Br(6)	94.84(14)		
Br(4)–Pb(2)–Br(6)	106.58(13)		
Br(8)–Pb(2)–Br(6)	82.54(12)		

Symmetry codes:(i): +x, 2−y, −1/2+z; (ii) +x, 2−y, 1/2+z; (iii) −1+x, +y, +z; (iv) 1+x, +y, +z.

**1*Rac***-370 K			
Br(1)–Pb(1)–Br(3)	85.61(6)	Pb(1)–Br(1)	2.9768(17)
Br(1)–Pb(1)–Br(4)	173.91(5)	Pb(1)–Br(2)	2.9669(7)
Br(1)–Pb(1)–Br(5)	94.04(6)	Pb(1)–Br(3)	3.0042(6)
Br(2)–Pb(1)–Br(1)	86.92(7)	Pb(1)–Br(4)	3.0567(17)
Br(2)–Pb(1)–Br(3)	171.26(6)	Pb(1)–Br(5)^{i}	2.9375(17)
Br(2)–Pb(1)–Br(4)	87.06(7)	Pb(1)–Br(5)	3.0951(17)

Br(2)–Pb(1)–Br(5)	81.56(4)
Br(2)–Pb(1)–Br(4)	100.46(6)
Br(3)–Pb(1)–Br(5)	103.47(4)
Br(4)–Pb(1)–Br(5)	84.12(5)
Br(5)^{i}–Pb(1)–Br(1)	90.36(6)
Br(5)^{i}–Pb(1)–Br(2)	87.31(7)
Br(5)^{i}–Pb(1)–Br(3)	88.21(5)
Br(5)^{i}–Pb(1)–Br(4)	90.32(6)
Br(5)^{i}–Pb(1)–Br(5)	167.78(4)
Pb(1)–Br(2)–Pb(1)^{i}	168.23(11)
Pb(1)^{iii}–Br(3)–Pb(1)	177.5(10)
Pb(1)^{iv}–Br(5)–Pb(1)	167.44(8)

Symmetry codes:(i) +x, 1−y, 1/2+z; (ii) 2−x, +y, 1/2−z; (iii) 1−x, +y, 1/2−z; iv +x, 1−y, −1/2+z.

Supplementary Table 4. Polynomial fitting equations and fitting parameters.

Equation	$y = Intercept + B_1*x + B_2*x^2$
Plot	T_C
Weight	No Weighting
Intercept	491.12622 ± 24.15133
B_1	−476.3049 ± 66.38703
B_2	420.97902 ± 44.06747
Residual Sum of Squares	83.30931
R-Square (COD)	0.98912
Adj. *R*-Square	0.9864

Supplementary Table 5. Summary of phase transition temperature (T_C), enthalpy change (ΔH), entropy change (ΔS), and orientation number (N) of $(S\text{-3AMP})_x(R\text{-3AMP})_{1-x}PbBr_4$ (x = 0.5 – 1).

EFE	T_C (heating) / K	ΔH / J/g	ΔH / kJ/mol	ΔS / J/(mol·K)	N
0.5	359.1	4.9	6.3	17.5	8.21
0.55	359.4	4.9	6.3	17.5	8.21
0.60	356.4	4.8	6.2	17.4	8.11
0.65	355.3	3.9	5.0	14.1	5.45
0.70	359.4	3.4	4.4	12.2	4.34
0.75	370.5	4.7	6.0	12.7	4.61
0.80	383.2	6.0	7.7	20.1	11.22
0.85	393.5	7.3	9.4	23.9	17.72
0.90	405.5	8.0	10.	25.4	21.22
0.95	419.0	9.0	11.6	27.7	27.99
1	432.7	10.3	13.2	30.1	37.35
